# Case Report: Decade-delayed thyroid metastasis with cervical lymph node involvement from clear cell renal cell carcinoma: diagnostic pitfalls of cytologic-radiologic discordance

**DOI:** 10.3389/fonc.2026.1878908

**Published:** 2026-06-12

**Authors:** Seok-Kyung Kang, Miri Ryu, Seungju Lee

**Affiliations:** Department of Surgery, Research Institute for Convergence of Biomedical Science and Technology, Pusan National University Yangsan Hospital, Pusan National University School of Medicine, Yangsan, Gyeongnam, Republic of Korea

**Keywords:** clear cell renal cell carcinoma, core needle biopsy, cytologic-radiologic discordance, fine-needle aspiration cytology, thyroid metastasis

## Abstract

**Background:**

Renal cell carcinoma (RCC), particularly the clear cell subtype, demonstrates a high metastatic potential and the capacity for late recurrence many years after nephrectomy. Although thyroid metastasis is uncommon, RCC is among the most frequently reported primary malignancies giving rise to secondary thyroid tumors. Distinguishing metastatic RCC from primary thyroid neoplasm is challenging on ultrasound and fine-needle aspiration cytology (FNAC) alone.

**Case presentation:**

A 55-year-old man with a history of clear cell RCC treated with radical nephrectomy 10 years earlier presented with a right thyroid nodule. Initial FANC at an outside institution demonstrated atypia of undetermined significance (AUS), and repeat FNAC at our institution yielded oncocytic follicular cells, despite progressively suspicious imaging findings. FNA of a right level III node was negative for malignancy, and lymph node washout thyroglobulin levels were low. The patient ultimately underwent right hemithyroidectomy with central and lateral neck dissection. Histopathology revealed metastatic clear cell RCC involving the thyroid and cervical lymph nodes, confirmed by immunohistochemistry showing positivity for CD10, vimentin, and carbonic anhydrase IX (CAIX), and negativity for thyroglobulin, TTF-1, and calcitonin.

**Conclusion:**

This case highlights the diagnostic challenges posed by indeterminate cytology and imaging-pathology discordance in late-presenting RCC thyroid metastasis. In patients with a prior history of RCC, new thyroid nodules or cervical lymphadenopathy should raise suspicion for metastatic disease even many years after initial treatment. Definitive diagnosis relies on histopathology and immunohistochemistry. When cytologic-radiologic discordance persists, early consideration of core needle biopsy is warranted. Management requires a multidisciplinary approach, with surgical resection of isolated metastatic disease integrated within an individualized, sequential systemic therapy strategy.

## Introduction

Renal cell carcinoma (RCC) accounts for the majority of malignant renal tumors in adults, with the clear cell subtype representing the most common histologic subtype and demonstrating a marked propensity for distant metastasis (1–3). Approximately 20-30% of patients present with metastatic disease at initial diagnosis (4, 5), and late metastases may occur many years or even decades after radical nephrectomy. Although the lung, bone, and liver are the most frequent metastatic sites, atypical organs such as the thyroid gland may also be involved (1, 6, 7).

Among secondary thyroid malignancies, RCC is consistently reported as one of the most frequent primary sources (1, 8–10). Clinically, metastatic RCC in the thyroid may present as a solitary nodule, multinodular goiter, or painless neck mass. Distinguishing metastatic RCC from primary thyroid carcinoma or benign nodular disease is often challenging based on ultrasound and fine-needle aspiration cytology (FNAC) alone (8–11). Definitive diagnosis frequently relies on histopathology and immunohistochemistry (IHC), with a characteristic profile of CD10, vimentin, carbonic anhydrase IX (CAIX), and RCC marker positivity and negativity for thyroglobulin and TTF-1 supporting the diagnosis (1, 2, 10–13).

We report a case of thyroid and cervical lymph node metastases detected 10 years after radical nephrectomy for clear cell RCC, with serial indeterminate cytology despite progressive radiologic suspicion. This case illustrates not only the diagnostic challenges of cytologic-radiologic discordance and the potential role of core needle biopsy, but also exemplifies the multimodal, sequential treatment approach that characterizes modern management of metastatic RCC.

## Case presentation

**Figure 1:**
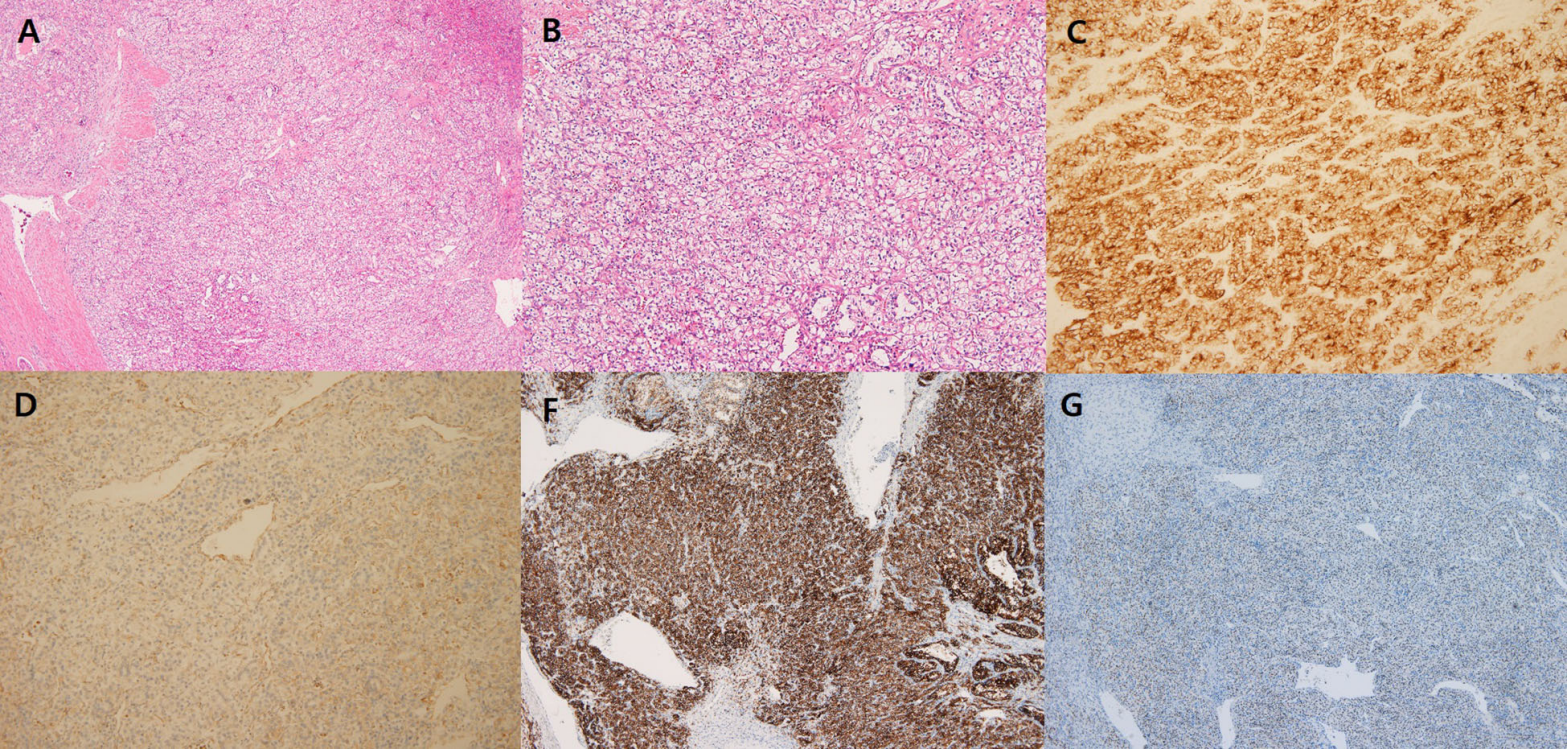
Histopathologic findings of primary renal tumor. On hematoxylin and eosin (H&E) staining, the tumor with abundant clear cytoplasm arranged in compact nests [**(A)**, ×40; **(B)**, ×100]. Immunohistochemistry demonstrates CD 10 positivity [**(C)**, ×100], vimentin positivity [**(D)**, ×100], CAIX positivity [**(E)**, ×40], and PAX-8 positivity [**(F)**, ×40].

A 55-year-old man presented with an incidentally noted right thyroid nodule. Ten years earlier (January 2014), he had undergone laparoscopic radical nephrectomy for clear cell RCC (**Figure 1**). The primary tumor (7.4 × 5.6 × 4.5 cm, lower pole) was pathologically staged as pT3aN0M0 (regional lymph nodes 0/5; AJCC 8^th^ edition) with Fuhrman nuclear grade 2/4 (corresponding to WHO/ISUP grade 2). The pT3a designation reflected invasion into the renal sinus fat and a large muscular vein, without perinephric fat involvement or lymphovascular invasion. Sarcomatoid features were absent, and surgical margins were uninvolved.

Subsequently (September 2014), video-assisted thoracoscopic (VATS) wedge resection of a solitary pulmonary metastasis was performed. An L4 vertebral metastasis was identified and treated with palliative radiotherapy (January-February 2015). In November 2015, suspected pancreatic metastasis was newly identified on imaging, and first-line systemic therapy with pazopanib, a tyrosine kinase inhibitor (TKI), was initiated on November 26, 2015 and continued until November 2024. Second-line therapy with everolimus (mTOR inhibitor) was subsequently administered from March 2025 to November 2025.

**Figure 2:**
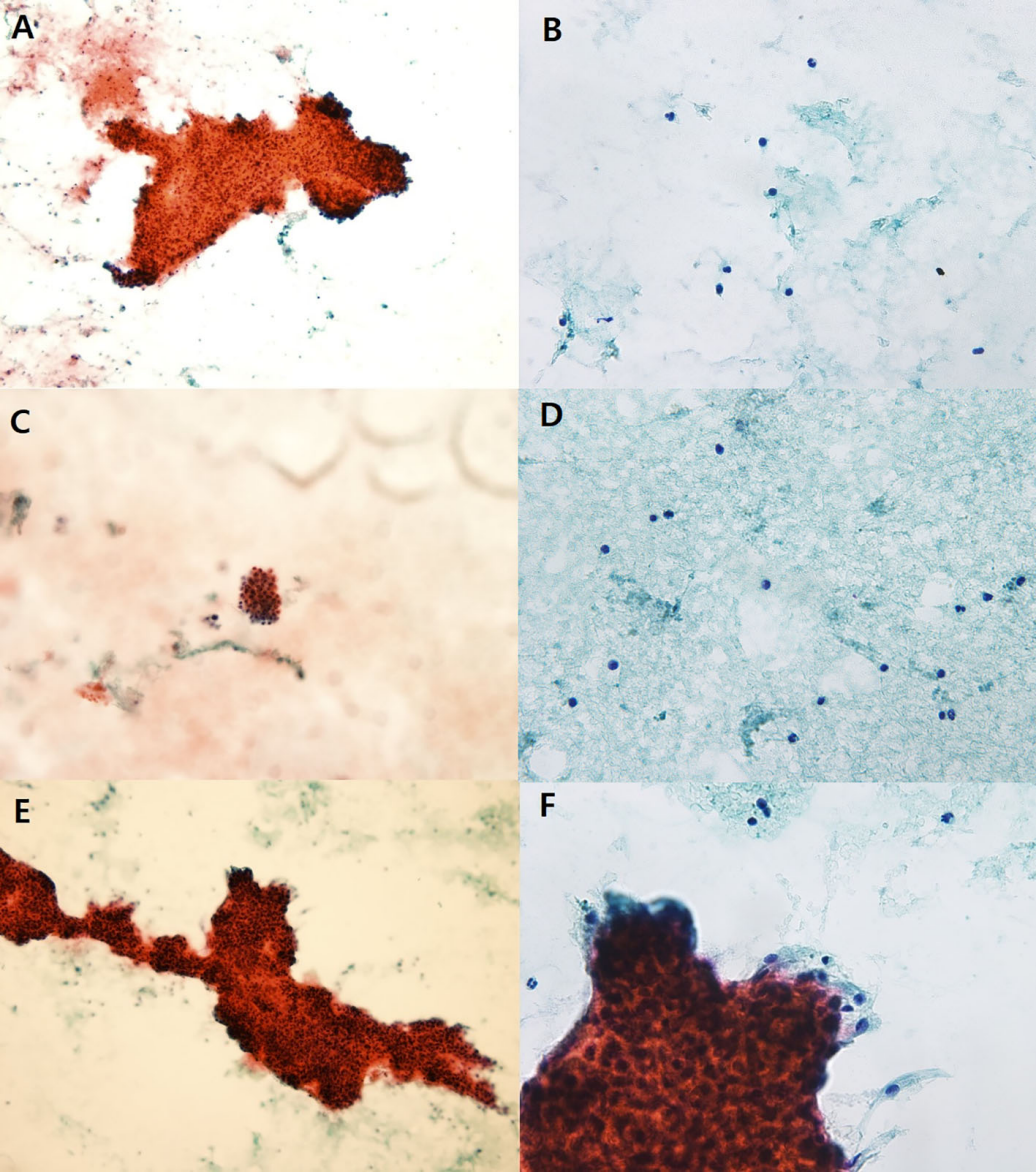
Fine-needle aspiration cytology findings. Thyroid FNA shows oncocytic follicular cells arranged in sheets and clusters with lymphohistiocytes in the background [**(A)**, ×40; **(B)**, ×400]. FNA of the ipsilateral level III cervical lymph node demonstrates inflammatory cells in clusters [**(C)**, ×40; **(D)**, ×400]. FNA of the level IV cervical lymph node reveals atypical cells in clusters mixed with inflammatory cells [**(E)**, ×40; **(F)** ×400].

On ultrasonography performed at an outside institution on July 16, 2024, a 3.6 × 3.4 × 3.8 cm heterogeneous nodule was identified in the right inferior pole, and FNAC yielded atypia of undetermined significance (AUS; Bethesda Category III). The patient was subsequently referred to our institution. Repeat fine-needle aspiration performed at our clinic three months later demonstrated oncocytic follicular cells (Bethesda Category III), and surveillance was elected (**Figures 2A, B**).

**Figure 3:**
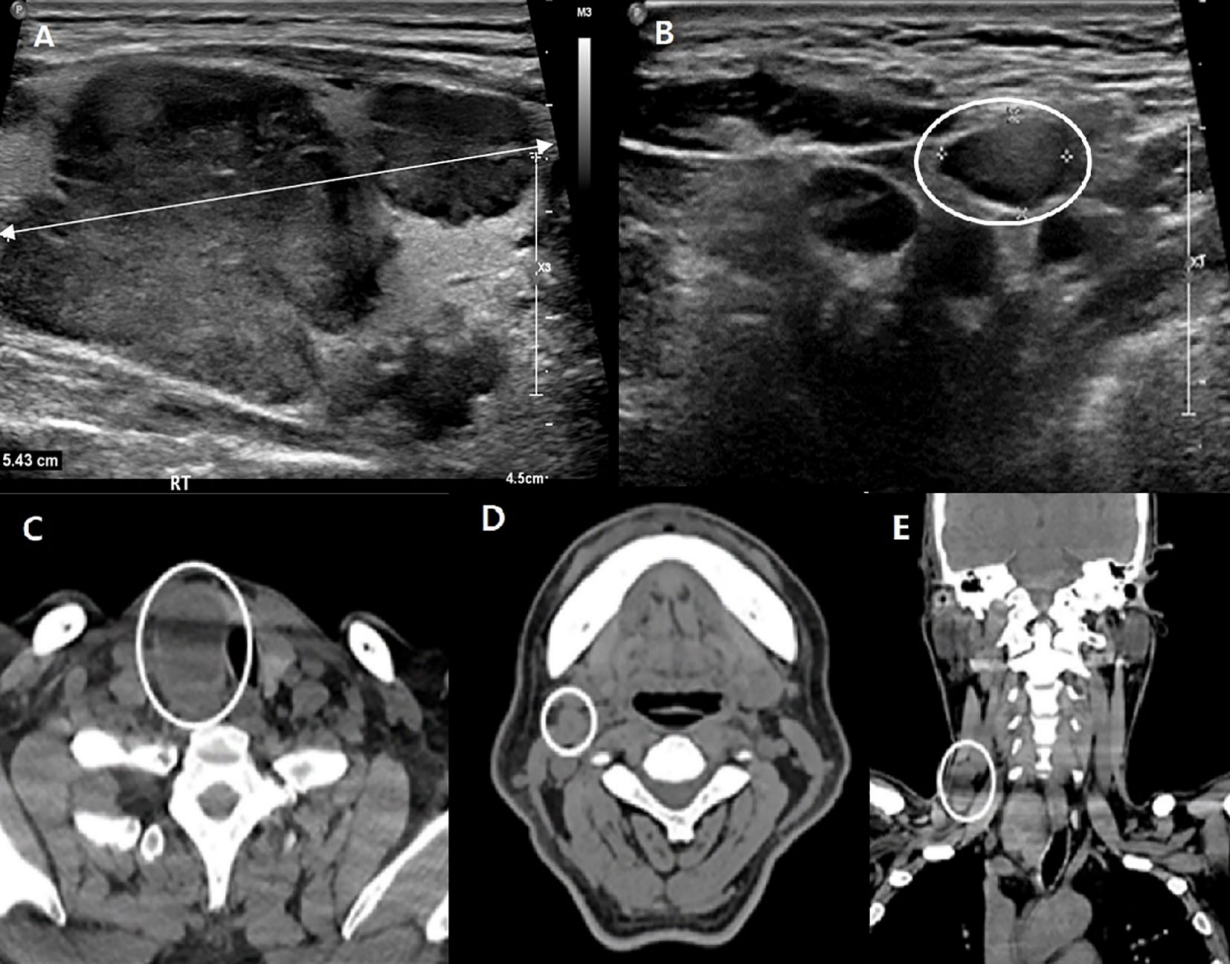
Radiologic findings of metastatic clear cell renal cell carcinoma involving the thyroid. **(A, B)** Thyroid ultrasound shows a large hypoechoic mas in the right thyroid lobe with satellite nodules and suspicious metastatic lymph nodes in level VI and III-IV. **(C, D, E)** Non-contrast-enhanced neck CT shows multiple right thyroid masses, suspicious lymph nodes in right neck level III-IV, and a 2.6cm infrathyroidal mass suggestive of metastatic extrathyroidal extension.

Six months later, follow-up ultrasound showed slight enlargement of the dominant right lower-pole nodule and a newly developed 1.2cm irregular hypoechoic TI-RADS 5 nodule. Suspicious lymph nodes were noted in right neck levels III and IV (**Figure 3**). FNA of a level III node was negative for malignancy (**Figures 2C, D**), with a low washout thyroglobulin level (0.58 ng/mL); observation was continued. At the subsequent 6-month follow-up, adjacent satellite nodules had increased in size. Imaging revealed a suspicious lesion in right level VI suggestive of extrathyroidal extension and multiple enlarged lymph nodes in levels III-IV on ultrasound and non-contrast-enhanced computed tomography (CT). FNAC of a right level IV node was again indeterminate (AUS) (**Figures 2E, F**), with very low washout thyroglobulin (0.15 ng/mL).

**Figure 4:**
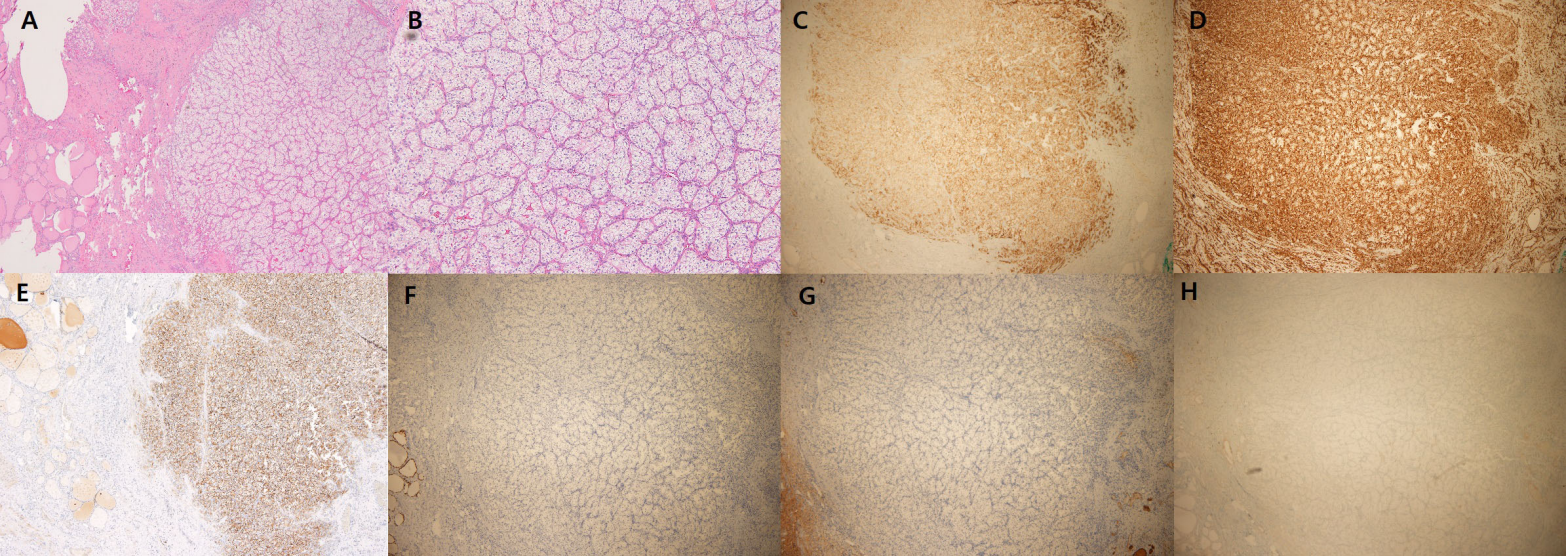
Histopathologic findings of metastatic clear cell renal cell carcinoma involving the thyroid. H&E staining shows tumor cells with clear cytoplasm in compact nests infiltrating the thyroid parenchyma [**(A)**, ×40; **(B)**, ×100]. IHC demonstrates positivity for CD10 [**(C)**, ×100], vimentin [**(D)**, ×100], and CAIX [**(E)**, ×40]. Staining is negative for TTF-1 [**(F)**, ×100], thyroglobulin [**(G)**, ×100], and calcitonin [**(H)**, ×100].

Given progressive radiologic suspicion and persistent cytologic indeterminacy, the patient underwent right hemithyroidectomy with central and right lateral neck lymph node dissection on January 8, 2026. Histopathology demonstrated 4.8 × 2.8 cm sized metastatic clear cell RCC involving the thyroid and right level VI (1/8) and level III-IV (2/6) lymph nodes. Tumor cells with abundant clear cytoplasm were arranged in compact alveolar nests infiltrating the thyroid parenchyma. The largest metastatic focus measured 2.5 cm; extranodal extension was present in one level III lymph node. IHC showed positivity for CD10, vimentin, and CAIX, and negativity for thyroglobulin, TTF-1, and calcitonin, consistent with metastatic RCC (**Figure 4**).

Following surgery, third-line systemic therapy with nivolumab (PD-L1 inhibitor) was initiated in February 2026 and continued through April 2026. Subsequent abdominal CT demonstrated enlargement of the pancreatic metastatic lesion, consistent with progressive disease (PD). Accordingly, fourth-line therapy with cabozantinib (TKI) was initiated in May 2026. The patient’s overall treatment course is summarized in **Supplementary Figure 1**.

## Discussion

Among secondary thyroid malignancies, RCC-particularly the clear cell subtype-is one of the most commonly reported primary tumors (1, 9, 10, 14). RCC is characterized by unpredictable metastatic behavior, including late dissemination many years after nephrectomy (3, 4, 6, 7, 11). The present case, with thyroid and cervical nodal metastases identified 10 years after nephrectomy and pulmonary metastasectomy, exemplifies this well-recognized biological pattern (1, 9, 11, 14). Notably, the primary tumor was pT3a at nephrectomy, indicating invasion beyond the renal capsule into the renal sinus fat and a large muscular vein-a feature associated with higher recurrence risk and capacity for late distant dissemination even following apparent locoregional control.

Diagnosis is frequently challenging. Metastatic RCC to the thyroid can mimic primary thyroid neoplasm both clinically and cytologically. Several studies have reported that metastatic lesions may be categorized as AUS or as follicular/oncocytic lesions within the Bethesda system (8, 10), as observed in our patients. Persistent discordance between suspicious imaging findings and indeterminate cytology should heighten clinical suspicion, particularly in patients with a prior history of RCC.

Although FNAC remains the first-line diagnostic modality, repeated indeterminate results in the context of progressive imaging abnormalities warrant consideration of core needle biopsy (CNB). CNB provides sufficient tissue for architectural assessment and immunohistochemical evaluation, facilitating more timely diagnosis of metastatic disease (15, 16). In our case, the approximately 18-month diagnostic interval from initial presentation to surgical confirmation underscores the potential value of earlier CNB in cytologic-radiologic discordant scenarios. Low lymph node washout thyroglobulin levels may suggest a non-thyroidal origin; however, this finding lacks absolute specificity, as poorly differentiated or non-secretory thyroid carcinomas may also demonstrate low values (17).

Definitive diagnosis relies on histopathologic examination and IHC. The characteristic immunoprofile of metastatic clear cell RCC includes CD10, vimentin, and RCC marker positivity, with negativity for thyroglobulin and TTF-1 (9, 11). CAIX in particular demonstrates diffuse membranous positivity in nearly all clear cell RCC cases and serves as an important confirmatory marker (13). In the present case, this immunophenotype effectively excluded primary differentiated and medullary thyroid carcinoma.

This case also illustrates the complexity of multimodal, sequential treatment in metastatic RCC. The patient received palliative radiotherapy for vertebral metastasis, followed by first-line pazopanib, second-line everolimus, and surgical management of the thyroid and cervical nodal disease-a decision justified by the relatively isolated locoregional recurrence at that juncture. Subsequently, third-line nivolumab was administered, with progression documented on pancreatic imaging, prompting a switch to fourth-line cabozantinib. Adjuvant therapy for metastatic RCC may include TKIs such as sunitinib and pazopanib, the mTOR inhibitor temsirolimus, interferon-alpha, sorafenib, and the immune checkpoint inhibitor nivolumab (18).In patients with isolated or limited thyroid and cervical nodal disease, thyroidectomy with or without neck dissection may provide durable local control (9, 11, 14, 19). In disseminated metastatic RCC, however, surgery is primarily diagnostic or palliative, and long-term prognosis depends largely on systemic therapy (7).

Although PD-L1 expression was not evaluated in the present case, assessment of PD-L1 status has been increasingly recognized as relevant in the context of immune checkpoint inhibitor therapy for metastatic RCC, as PD-L1 positivity may inform therapeutic sequencing decisions (7). This represents a limitation of our report, and retrospective PD-L1 testing will be attempted if archival tissue is sufficient.

The strengths of this report lie in its detailed longitudinal document of cytologic and radiologic findings over an approximately 18-month interval, and its demonstration of the role of IHC in resolving diagnostic uncertainty. It further illustrates a complex, multimodal treatment course spanning over a decade. The main limitations are (1): CNB was not performed during the period of cytologic-radiologic discordance, which may have accelerated diagnosis (2); PD-L1 staining were not performed on the thyroid metastasis specimen (3); the primary RCC slides are from archival tissue over a decade old, limiting the quality of histologic images available; and (4) generalizability is limited by the single-case design.

This case underscores that in patients with a history of RCC, new thyroid nodules and cervical lymphadenopathy-even many years after nephrectomy-should prompt strong consideration of metastatic disease. Cytologic findings, imaging features, washout thyroglobulin levels, and immunohistochemical results must be interpreted in conjunction with oncologic history and the ongoing systemic treatment trajectory to achieve accurate diagnosis and appropriate multidisciplinary management.

## Patient perspective

The patient provided written informed consent for publication of this case report and accompanying images. He reported that the prolonged diagnostic workup over 18-month interval was a source of significant uncertainty and anxiety. He expressed that clearer communication regarding the possibility of metastatic disease at earlier stages, in light of his prior RCC history, would have helped him prepare psychologically. He understood and accepted the rationale for surgical intervention and reported satisfactory recovery following the procedure.

## Data Availability

The raw data supporting the conclusions of this article will be made available by the authors, without undue reservation.
