# Supplementary figures and images for: Case Report: Decade-delayed thyroid metastasis with cervical lymph node involvement from clear cell renal cell carcinoma: diagnostic pitfalls of cytologic-radiologic discordance
Source: Front Oncol. 2026 Jun 12;16:1878908. doi: 10.3389/fonc.2026.1878908 (PMC13303188; doi:10.3389/fonc.2026.1878908)

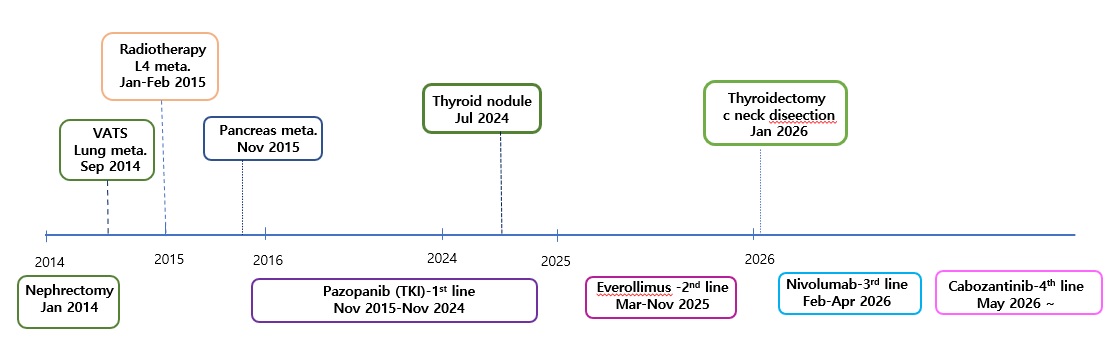

Supplement: Supplementary file 1 [file Image1.jpeg]
